# Extraction, Identification and Photo-Physical Characterization of Persimmon (*Diospyros kaki* L.) Carotenoids

**DOI:** 10.3390/foods6010004

**Published:** 2017-01-12

**Authors:** Khalil Zaghdoudi, Orleans Ngomo, Régis Vanderesse, Philippe Arnoux, Bauyrzhan Myrzakhmetov, Céline Frochot, Yann Guiavarc’h

**Affiliations:** 1 Laboratoire Réactions et Génie des Procédés, Université de Lorraine-CNRS, UMR 7274, 1 Rue Grandville, BP 20451, 54001 Nancy Cedex, France; khalilo.zg@gmail.com (K.Z.); orleansn@yahoo.fr (O.N.); philippe.arnoux@univ-lorraine.fr (P.A.); baur_86_86@mail.ru (B.M.); yann.guivarch@univ-lorraine.fr (Y.G.); 2 Laboratoire de Chimie Physique Macromoléculaire, Université de Lorraine-CNRS, UMR 7375, 1 Rue Grandville, BP 20451, 54001 Nancy Cedex, France; regis.vanderesse@univ-lorraine.fr; 3 Laboratoire d’Application de la Chimie aux Ressources et Substances Naturelles et à l’Environnement, Faculté des Sciences de Bizerte, Université de Carthage, Carthage 1054, Tunisie; 4 Laboratoire des Matériaux et Chimie Industrielle Inorganique, ENSAI—University of Ngaoundere, Ngaoundere 454, Cameroun

**Keywords:** persimmon, carotenoids, purification, singlet oxygen, quenching, porphyrin

## Abstract

Carotenoid pigments were extracted and purified from persimmon fruits using accelerated solvent extraction (ASE). Eleven pigments were isolated and five of them were clearly identified as all-*trans*-violaxanthine, all-*trans*-lutein, all-*trans*-zeaxanthin all-*trans*-cryptoxanthin and all-*trans*-β-carotene. Absorption and fluorescence spectra were recorded. To evaluate the potential of ^1^O_2_ quenching of the purified carotenoids, we used a monocarboxylic porphyrin (P1COOH) as the photosensitizer to produce ^1^O_2_. The rate constants of singlet oxygen quenching (Kq) were determined by monitoring the near-infrared (1270 nm) luminescence of ^1^O_2_ produced by photosensitizer excitation. The lifetime of singlet oxygen was measured in the presence of increasing concentrations of carotenoids in hexane. Recorded Kq values show that all-*trans*-β-cryptoxanthin, all-*trans*-β-carotene, all-*trans*-lycopene and all-*trans*-zeaxanthin quench singlet oxygen in hexane efficiently (associated Kq values of 1.6 × 10^9^, 1.3 × 10^9^, 1.1 × 10^9^ and 1.1 × 10^9^ M^−1^·s^−1^, respectively). The efficiency of singlet oxygen quenching of β-cryptoxanthin can thus change the consideration that β-carotene and lycopene are the most efficient singlet oxygen quenchers acting as catalysts for deactivation of the harmful ^1^O_2_.

## 1. Introduction

Carotenoids are some of the most widespread lipidic soluble pigments in nature; more than 750 naturally occurring carotenoids have been identified to date, but only a few are commercially available in a pure form and are expensive. They are produced as secondary metabolites by all photosynthetic organisms (plants, fungi, yeasts and non-photosynthetic bacteria) and are also accumulated from human and animal diets [1,2]. Carotenoids contribute to both organoleptic and nutritional properties of food and food by-products providing red, yellow or orange colors for fruits and vegetables. They are used as food colorants in foods and animal products such as egg yolk, butter, crustaceans, trout, salmon and shrimp [3]. They also contribute to the pigmentation of birds’ plumage [4]. Generally, most carotenoids can be derived from C40 tetraterpenoids polyene backbone including a system of conjugated double bonds in which the π electrons are effectively delocalized over the entire chain; the skeleton can carry cyclic ends groups with substitution of functional groups containing oxygen [5]. Through their structure, carotenoids are classified into two main groups: (i) carotenes also called carotenoid hydrocarbons, which only contain carbon and hydrogen; and (ii) xanthophylls or oxygenated carotenoids that may contain different functional groups (epoxy, methoxy, hydroxy, carbonyl and carboxyl acid groups) [6,7]. The most characteristic feature of carotenoids is light absorption in the high-energy zone of the visible region (400–500 nm) by the long conjugated double bond system. Such a conjugated chain structure is crucial for carotenoid function especially in photosynthetic organisms as well as for photo-protection in all living organisms [8]. Carotenoids are involved in photosystem assembly, where light harvesting provides protection from excess light absorption through energy dissipation and radical detoxification [9]. 

Besides their role in photosynthesis and photo-protection, carotenoids are involved in the prevention of several human diseases and represent part of the antioxidant defense system in humans [10]. They act as cardioprotective agents and prevent acute myocardial infarction [11], prevent the development of reactive oxygen species-mediated disorders related to oxidative stress and can be responsible for reducing the low-density lipoprotein (LDL) cholesterol level [12]. Since two decades, several prospective studies have also shown a correlation between the consumption of fruits and vegetables rich in carotenoids and a decreased risk of many types of cancer [13]. Among these malignant diseases, prostate cancer [14], liver tumors, skin tumorigenesis and breast carcinogenesis [15] can be cited. Each carotenoid can be particularly involved—for example, canthaxanthin suppresses human colon cancer cells proliferation [16], while astaxanthin exhibits inhibitory activity in relation to cancer development in urinary bladder [17] and colorectum [18].

As previously written, one of the carotenoid functions is the protection against damage mediated by light, and carotenoids can act as quenchers to prevent the formation of ^1^O_2_ in biological systems. Moreover, in the presence of photosensitizers (PS) such as bacteriochlorophyll, chlorophyll or protoporphyrin IX, which absorb light and generate ^1^O_2_, they can quench the triplet state energy from the sensitizer or from ^1^O_2_ [19]. Foote et al. were the first to carry out a comprehensive study of ^1^O_2_ quenching mechanisms by β-carotene [20]. Due to these ^1^O_2_ quenching properties, the use of carotenoids for anticancer photodynamic therapy (PDT) has been developed, particularly to design “photomolecular beacons” (PMB), which are composed of an energy acceptor and an energy donor connected one to the other by a link (usually a peptide or nucleotide sensitive to an endogenous stimulus). This concept has been first developed by Zheng’s team [21,22] and is a part of our current studies [23]. Briefly, the energy acceptor (carotenoid) and the donor (PS) are maintained sufficiently close to each other to allow a transfer of energy, and the carotenoid can quench ^1^O_2_ generation through PS triplet-state energy transfer and/or ^1^O_2_ scavenging. If a specific enzyme cleaves the peptidic or nucleosidic link the photophysical properties of the photosensitizer is restored and PDT can be applied.

The aim of this study was to try to find unusual carotenoids, which would have a potential application in PDT as ^1^O_2_ quenchers. Eleven carotenoids were extracted and purified from persimmon fruit cultivated in Tunisia and known for its richness in carotenoid pigments, using accelerated solvent extraction (ASE) [24]. After separation, the structure of five of the carotenoids was determined and their potential evaluated as singlet oxygen quenchers in hexane.

## 2. Materials and Methods

### 2.1. Chemicals

All-*trans*-β-carotene (Type II synthetic, purity >95%), lutein from marigold, β-cryptoxanthin (purity >97%), zeaxanthin (purity >95%), lycopene from tomato (purity >90%), butylhydroxytoluene (BHT, purity ≥99%) and triethylamine (TEA, purity ≥99%) were obtained from Sigma-Aldrich (Shnelldorf, Germany). High-performance liquid chromatography (HPLC) organic solvents were of analytical grade: methanol (MeOH), absolute ethanol (EtOH), chloroform and hexane were from Carlo Erba (Val-de-Reuil, France), methyl-tert-butyl-ether (MTBE) was from Fisher Scientific (Loughborough, UK), petroleum ether (PE) was from VWR Prolabo (Fontenay-sous-Bois, France). Ultrapure water was obtained from a purified water system Arium^®^ 611UV from Sartorius (Göttingen, Germany) with a resistivity of 18.2 MΩ*cm. Sodium chloride (NaCl, purity >99%) and potassium hydroxide (KOH, purity >99%) were obtained from VWR Prolabo (Fontenay-sous-Bois, France), and activated magnesium silicate Florisil^®^ at 100–200 mesh (Sigma-Aldrich, Shnelldorf, Germany). Liquid Nitrogen was from Air Liquide (Nancy, France). The synthesis of 5-(4-carboxyphenyl)-10,15,20-triphenyl porphyrin (P1COOH) has been described elsewhere [25].

### 2.2. Fruit Materials

The present study focuses on 27 persimmon fruit (*Diospyros kaki* L.) cultivated in Tunisia (persimmon known under the appellation “Krima”); persimmon fruits were harvested at full maturity from three selected trees. The choice of persimmon as extraction matrix was built on earlier observations from previous investigation on carotenoid profiling from *Prunus persica* (peach), *Prunus armeniaca* (apricot) and *Diospyros kaki* (persimmon) fruits, largely produced in Tunisia [24], which shows that persimmon hole fruit is a rich source of carotenoids (xanthophylls and carotenes). Persimmon carotenoid profiles gave a large pigment range to assay; however, commercial carotenoids are still (i) rare; (ii) expensive; and (iii) with doubtful purity. 

### 2.3. Sample Preparation and Characterization

Selected fruits were immediately washed with distillated water and placed in hermetical polystyrene boxes in which we added some liquid nitrogen in order to prevent oxidation reactions, before to be stored at −20 °C. Whole fruits (flesh and peel, 5 kg) were manually sliced into approximately 2 g pieces, frozen at −80 °C, and at once freeze-dried during 48 h with a pilot CryoTec freeze-dryer (Saint-Gély-du-Fesc, France) with a separated cold trap at −70 °C, and plate temperature set to −20 °C at a working pressure of 15 Pa. Freeze-dried fruit pieces were then placed in a desiccator containing P_2_O_5_ in order to prevent water sorption. After freeze-drying, fruit pieces were immediately grounded with a Moulinex coffee grinder (Moulinex, Ecully, France). A small amount of liquid nitrogen were added to prevent oxidation during grinding. Powders were stored in a desiccator containing P_2_O_5_ in order to prevent water sorption, and their residual water content was measured after 24 h at 105 °C. Particle size distribution of fruit powders was measured through dynamic light scattering with a Malvern Mastersizer 2000 apparatus (Malvern Instruments Ltd, Malvern, UK) in absolute ethanol.

### 2.4. Accelerated Solvent Extraction of Carotenoids

Carotenoid extraction experiments were carried out in a static mode with a Dionex ASE 350 extractor (Salt Lake City, UT, USA) using 12 stainless steel cells of 22 mL volume. Amber vials (60 mL) were used for extract collection. Common extraction parameters were as follows: (i) fruit powder (2 g) was loaded into the cell, and residual cell volume was partially filled with 2 mm glass beads; (ii) the cell was filled with solvent to a pressure of 1500 psi (103 bars); (iii) heat was applied for an initial heat-up time of 5 min; (iv) static extraction with all system valves closed was performed with a 5 min cycle time; (v) the cell was rinsed with 60% of the cell volume with extraction solvent; (vi) the solvent was purged from the cell with N_2_ gas for 60 s, and (vii) the system was depressurized. Extraction solvents, MeOH and tetrahydrofuran (THF), were degassed for 30 min by ultrasonic bath Sonoclean^®^ Labo Moderne (Paris, France) before use. Florisil (2 g) was added as a dispersing agent for persimmon powder. Extraction parameters were set according to the common optimal conditions found in our previous study through an experimental design [24] as follows: temperature was set at 40 °C, 5 min static time extraction (one cycle), and binary extraction solvent (MeOH/THF, 20/80, *v*/*v*) under 103 bars.

### 2.5. Preparation of ASE Carotenoids Extracts for HPLC Semi-Preparative Purification

Pressurized liquid carotenoid extracts were prepared according to the method described in [24] and were conducted under limited light. At first, the ASE extract was homogenized with a vortex (Scientific Industries Inc., New York, NY, USA) at 13,500 rpm during 1 min before being filtered under vacuum through an AP 25 glass fiber filter disc of 2 µm porosity (Millipore, Darmstadt, Germany). Then, 10 mL of the filtrate was added to 10 mL of 10% (*w/v*) aqueous sodium chloride solution and carotenoids were transferred to a 10 mL petroleum ether phase containing 0.1% BHT (w/v) using a liquid–liquid extraction repeated at least three times in order to achieve a total transfer of carotenoids toward the petroleum ether phase. Organic phase was subsequently washed three times with 10 mL of ultrapure water in order to remove residual NaCl traces under neutral pH. Petroleum ether was then evaporated at 30 °C under nitrogen flow using a Turbo Vap^®^ LV (Biotage AB, Uppsala, Sweden). The residue was rapidly dissolved in 5 mL of mobile phase (MeOH/MTBE, 1/1, v/v) containing 0.1% BHT (*w/v*) and filtered through 0.45 µm polyvinylidene fluoride (PVDF) syringe filters (Pall Life Sciences, Ann Arbor, PN, USA). Then, the filtered solution was mixed with 2.5 mL of MeOH containing 0.1% BHT (*w/v*) and saponified with 2.5 mL of 20% (*w/v*) methanolic KOH solution under nitrogen, in the dark, for 1 h, at room temperature. Finally, the saponified sample was injected for the semi preparative assay.

### 2.6. Semi Preparative HPLC Reversed Phase Carotenoid Purifications

Carotenoid separation was performed by reverse phase chromatography on a preparative Gilson HPLC GX-271 system consisting of a GX-271 Handler injector/collector equipped with a single 402 syringe pump, a 322 Semi-preparative HPLC pump and a 156 DAD detector at 450 nm from Gilson (Middleton, WI, USA) controlled by Trilution LCv2.1 software (Gilson, Middleton, WI, USA) We used a 250 length × 4.6 mm inside diameter S-5 µm Yamamura Chemical (YMC) C30 semi preparative column (Yamamura Chemical Europe GmbH, Schöttmannshof, Germany) coupled to a 10 × 4.0 mm inside diameter (ID) S-5µm guard cartridge semi preparative column (ImChem, Versailles, France). The purification assay was performed at 25 °C. The mobile phase consisted of a MeOH gradient containing 0.05 M ammonium acetate (A), and MTBE (B). Both solvents A and B contained 0.1% BHT (*w/v*) and 0.05% of triethylamine (TEA) (*w/v*). The flow rate was 4.0 mL/min and the injected volume was 3.0 mL. The gradient profile of the mobile phase was set as follows: a linear increasing gradient from 2% B to 20% B in 60 min; a linear increasing gradient from 20% B to 95% B in 20 min; a linear gradient at 95% B in 10 min, decreasing from 95% B to 2% B in 5 min; and a linear gradient at 2% B in 5 min. The column was equilibrated for 10 min in the starting conditions before each injection. Before each batch of HPLC semi-preparative purification, the stabilization time of the column was 30 min, and a blank (MeOH/MTBE, 1/1, *v/v*) was injected. The same pooled fraction of carotenoids was collected and then concentrated at 30 °C under nitrogen flow using a Turbo Vap^®^ LV (Biotage AB, Uppsala, Sweden) in amber glass vials prior to characterization.

### 2.7. Identification of Unknown Carotenoids

In order to identify additional carotenoids and compare the fruits carotenoids profiles, liquid chromatography-mass spectrometry (LC-MS) runs were performed in positive atmospheric pressure chemical ionization (APCI) mode with a Shimadzu LC-MS-2020 liquid chromatograh-mass spectrometer (Shimadzu, Marne-la-Vallée, France). A scanning rate of 2143 u/s was used in the range 50–2000 amu. Nebulizing gas flow was fixed at 1.5 mL/min. Interface voltage and temperature were 4.5 kV and 250 °C, respectively. The gradient profile of the mobile phase was set as follows: linear increasing gradient from 5% B to 30% B in 30 min; linear increasing gradient from 30% B to 95% B in 20 min; and linear decreasing gradient from 95% B to 5% B in 10 min. The column was equilibrated for 5 min at the starting conditions before each injection.

### 2.8. Photophysical Properties

Absorption spectra were recorded on a PerkinElmer (Lambda EZ 210, Shelton, CT, USA) double beam UV-visible spectrophotometer. Fluorescence spectra were recorded on a Horiba Jobin Yvon Fluorolog-3 (FL3-222) spectrofluorometer equipped with a 450 W Xenon lamp, a thermostated cell compartment (25 °C), an R928 (Horiba, Longjumeau, France) UV-visible photomultiplier and a liquid nitrogen cooled InGaAs infrared detector (DSS-16A020L Electro-Optical System Inc., Phoenixville, PA, USA). The excitation spectrometer is a SPEX double grating monochromator (1200 grating/mm blazed 330 nm). The fluorescence was measured by the UV-visible detector through a SPEX double grating monochromator (600 grooves/mm blazed 1 nm). Singlet oxygen lifetime measurements of 5-(4-carboxyphenyl)-10,15,20-triphenyl porphyrin (P1COOH) with or without carotenoids were performed on a TEMPRO-01 spectrophotometer (Horiba Jobin Yvon, Palaiseau, France) composed of a pulsed diode excitation source SpectraLED-415 emitting at 415 nm, a cuvette compartment, a Seya–Namioka type emission monochromator (600–2000 nm) and a H10330-45 near-infrared photomultiplier tube with a thermoelectric cooler (Hamamatsu) as the detection device. The system was monitored by a single photon counting FluoroHub-B controller and the DataStation and DAS6 software (version, Horiba Jobin Yvon, City, US State abbrev if applicable, Country).

The Stern–Volmer equation was used to evaluate the constant quenching *K_q_* of ^1^O_2_ by the different carotenoids τ_0_/τ = 1 + *K_q_*(Car), where τ_0_ is the ^1^O_2_ lifetime after excitation of P1COOH in hexane without carotenoid, τ is the ^1^O_2_ lifetime after excitation of P1COOH in hexane with carotenoid, and (Car) is the carotenoid concentration. The concentration of P1COOH was kept constant at 2.25 × 10^−6^ mol/L, whereas the concentration of carotenoids varied between 0 and 1.5 × 10^−3^ mol/L. The ratio of (Car)/(P1COOH) was increasing from 0 to 670. 

## 3. Results and Discussion

### 3.1. Particle Size Distribution of Persimmon Freeze-Dried Powder

**Figure 1:**
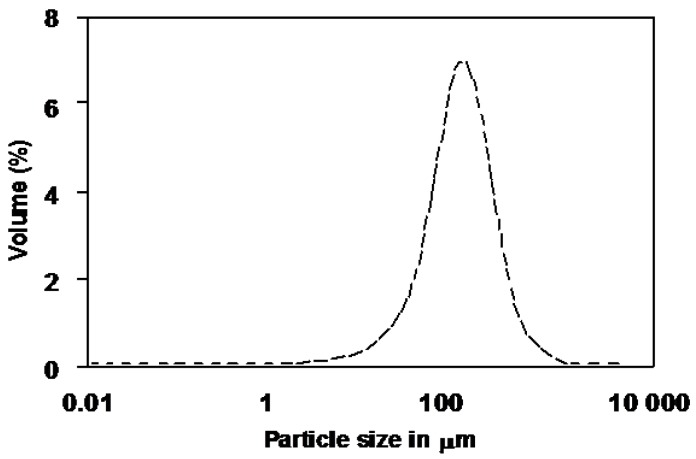
Particle size distribution of persimmon freeze dried powder.

Figure 1 shows the particle size distribution of persimmon freeze dried powder used for carotenoid extraction. All the particles present a diameter below 500 µm, which is currently recognized as appropriate for good ASE extractions [26]. The particle size of the freeze dried matrix is indeed a key factor in extraction of bioactive analytes from complex food matrices. The efficiency of the extraction depends on the analyte to be extracted, the nature of the sample matrix and the analyte location within the matrix [27]. Particle size, together with pressure and temperature in the ASE process, is a critical factor to enhance extraction efficiency. Reducing the particle size of the solid matrix increases the mass transfer surfaces with the extraction solvent. Reducing the particle size also facilitates solvent diffusion through the matrix pores and the partitioning of carotenoids between the matrix and the extraction fluid, thus leading to accelerated extraction. 

### 3.2. Semi-Preparative HPLC Purification and Identification of Carotenoids in the ASE Extract

**Figure 2:**
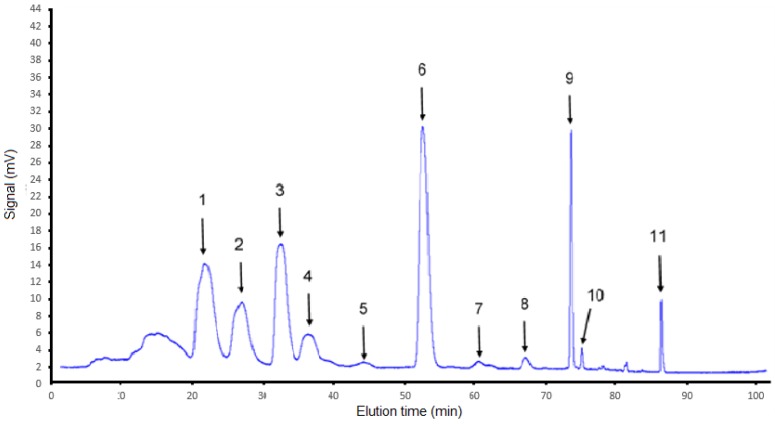
C_30_ semi preparative chromatogram of carotenoids extracted from persimmon.

Analytical HPLC separation conditions of persimmon carotenoids were well described previously [24]. Figure 2 shows the ASE carotenoid extract chromatogram. Eleven carotenoids were detected due to their absorption at 450 nm and the retention times were given in Table 1. Their masses were calculated and are also found in Table 1. Five of the 11 purified carotenoids were identified using commercial standards and LC-MS (APCI positive mode), that is to say compounds 1, 2, 3, 6 and 9 as violaxanthin, lutein, zeaxanthin, β-cryptoxanthin and β-carotene, respectively [24]. 

As can be seen in Table 1, after fraction collections corresponding to each peak, eleven peaks were detected and identified as carotenoids based (i) on their retention time (as compared to commercial standards when available) and (ii) on their spectral characteristics after chromatographic solvent evaporation and solubilization in petroleum ether (λ_max_, fine spectral data as compared to other spectral data from the literature as described in Table 1 footnotes). In each case, *m/z* values were also determined from liquid chromatography coupled to mass spectrometry (LC-MS) analysis. The six first peaks identified as carotenoids logically correspond to polar carotenoids (xanthophylls). The higher the number of hydroxyl and/or epoxy groups, the shorter the retention time. While peak 7 is not clearly identified (mixture), logically, the last four peaks were identified as carotenes (no polar groups such as OH or epoxy groups). *Cis*-isomeric forms of carotenoids can easily be detected from the presence of a first λ_max_ in the UV domain around 330 nm (therefore, *cis*-isomeric forms are characterized by 4 λ_max_ instead of 3 λ_max_ only for *trans*-isomeric forms). Each peak identification in Table 1 is explained in detail below.

- *All* trans*-violaxanthin (peak 1**):* [M + H]^+^ *m/z*, λ_max_, absence of *cis*-peak and a short retention time are in perfect agreement with the identification of all-*trans*-violaxanthin, which contains two hydroxyl and two epoxy groups. All-*trans*-violaxanthin had already been identified in persimmon [27,30]. However the % (III/II) observed here was 50 instead of 98, which may be due to the origin of the petroleum ether that we used. It should be noted that petroleum ether is not a well-defined molecule but a mixture of C5 and C6 hydrocarbons.
- *All* trans*-lutein (peak 2)*: [M + H]^+^ *m/z*, λ_max_, absence of *cis*-peak and a short retention time similar to that of the standard are in perfect agreement with the identification of all-*trans*-lutein, which contains two OH groups and should have a slightly longer retention time than violaxanthin. All-*trans*-lutein had already been identified in persimmon [30] However, the % (III/II) observed here was 25 instead of 60, which may be due to the origin of the petroleum ether that we used.
- *All* trans*-zeaxanthin (peak 3)*: the characteristics found fit the data perfectly from the literature [28]. The retention time is also in agreement with that of the standard.
- *Not identified (peak 4):* The [M + H]^+^ *m/z* value of 601 indicates a xanthophyll with four oxygens. The fact that, despite these fours oxygens, the fact that this xanthophyll has a longer retention time than lutein, and even zeaxanthin, indicates that this is a *cis*-form of xanthophyll and indeed we observed a slight *cis*-peak at 330 nm and an associated % A_B_:A_II_ of 14%.
- *Suspected 5,6-epoxy-α-carotene (peak 5):* [M + H]^+^ *m/z*, λ_max_, the absence of a *cis*-peak, as well as the % III:II, all suggest the presence of 5,6-epoxy-α-carotene.
- *All-*trans*-β-cryptoxanthin (peak 6)*: the characteristics found fit the data from the literature [29]. The retention time is also in agreement with that of the standard. This xanthophyll is known to be the most prevalent carotenoid in persimmon.
- *Unidentified (peak 7):* [M + H]^+^ *m/z* values of 553 and 537 indicate a mixture of a single-oxygenated xanthophyll and of one carotene, with retention times that do not allow separation by the preparative chromatography fraction collector. A very slight increase in absorbance is observed at 330 nm, which could indicate a *cis*-form. It is not possible to be more precise.
- Cis*-isomer of β-carotene, supposed position 13 (peak 8)*: the mass indicates that this is a carotene and the presence of a slight peak at 330 nm indicates a *cis*-form of carotene. λ_max_ as well as the % (III:II) indicates that it is most likely a *cis*-isomer of β*-*carotene. However, the % A_B_:A_II_ is lower than that found by De Rosso [29], but it should be noted that they used a different solvent. Therefore, this position 13 of *cis*-isomerization can only be presumed.
- *All-*trans*-β-carotene (peak 9)*: the characteristics found fit the data from the literature perfectly [28]. The retention time is also in agreement with that of the standard.
- Cis*-isomer of β-carotene, supposed position 9 (peak 10)*: the mass indicates that this is a carotene and the presence of a slight peak at 330 nm indicates a *cis*-form of carotene. λ_max_ as well as the % (III:II) indicates that it is very likely a *cis*-isomer of β*-*carotene. However, the % A_B_:A_II_ is higher than that found by De Rosso [29], but it should be noted that they used a different solvent. This % should also be lower than that of 13-*cis*-β-carotene, which is not the case. Thus, position 9 of the *cis*-isomerization can only be presumed.
- *All-*trans*-lycopene (peak 11)*: all-*trans* lycopene was precisely identified, especially due to its characteristic set of λ_max_ values [28].

### 3.3. Details on UV-Visible and Fluorescence Spectra of Purified Carotenoids

**Figure 3:**
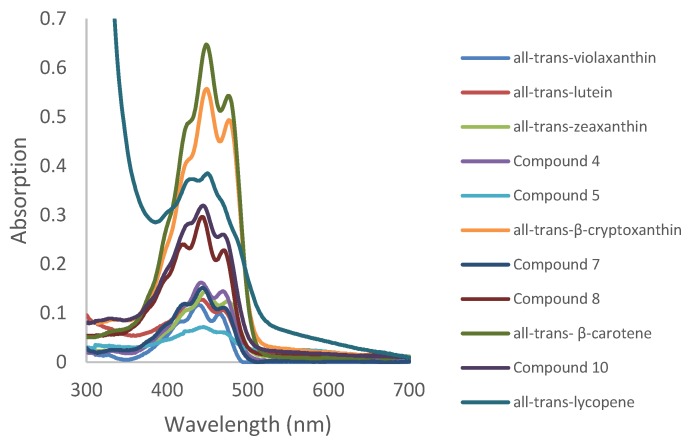
Absorption spectra of all the purified carotenoids in petroleum ether.

**Figure 4:**
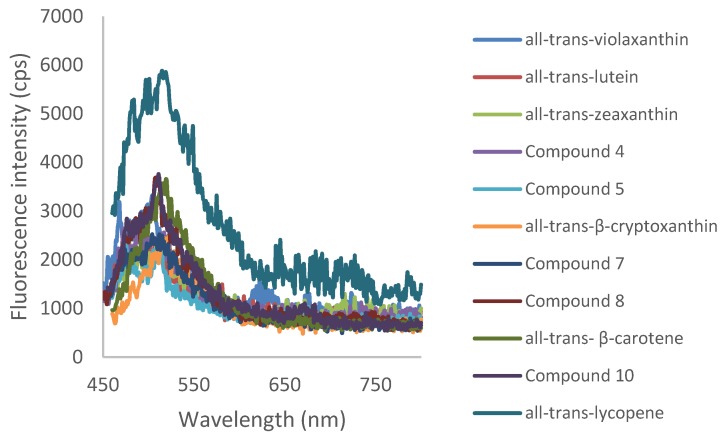
Fluorescence spectra of all compounds in petroleum ether (λ_ex_ varying from 439 to 451 nm).

Figure 3 and Figure 4, respectively, show the Ultraviolet-Visible (UV-Vis) absorption spectra and fluorescence spectra of carotenoids extracted and purified from persimmon fruits in petroleum ether. As explained after Table 1, fine UV-Vis spectral characteristics were derived from Figure 3. Regarding fluorescence spectra, different excitation wavelengths were chosen from 439 nm to 451 nm in order to excite in a maximum of absorption. It is often considered that the carotenoids are non-fluorescent compounds. In our conditions, we could nevertheless detect a very weak fluorescence with a band centered at around 500 nm. 

### 3.4. Singlet Oxygen Quenching by Carotenoids

**Figure 5:**
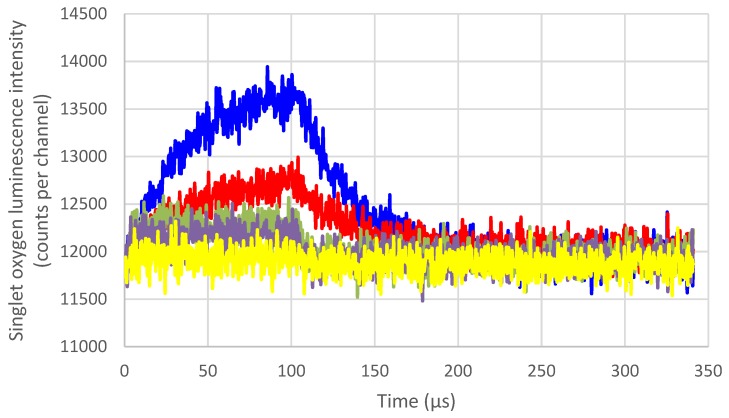
^1^O_2_ decay of P1COOH without all-*trans*-β-carotene and with increasing amounts of all-*trans*-β-carotene (Molar concentration), λ_exc_ = 408 nm. *c* = 2.25 × 10^−6^ mol/L in hexane. The ratio of (Car)/(P1COOH) was increasing from 0 to 670 (blue: 0 mol/L, red: 4 × 10^-7^ mol/L, green: 4 × 10^-5^ mol/L, pink: 2 × 10^-4^ mol/L, yellow: 4 × 10^-4^ mol/L).

To evaluate the potential of ^1^O_2_ quenching, we used a porphyrin (P1COOH) to produce ^1^O_2_ and we added different amounts of carotenoids. We recorded the ^1^O_2_ decay of P1COOH without all-*trans*-β-carotene and with increasing amounts of all-*trans*-β-carotene (Figure 5). 

Due to this decay, the Stern–Volmer plot can be built and the quenching constant evaluated. Data are already available in the literature and are reported in Table 2, but no evaluation of the quenching constants in hexane has been assessed to date. Our results are reported in Table 3. 

Some photosensitizers have been reported in the literature to evaluate the fluorescence potential or ^1^O_2_ quenching of various carotenoids (Table 2). Dreuw et al. [31] studied chlorophyll fluorescence quenching by xanthophylls using time-dependent density-functional theory and configuration interaction singles. Naqvi et al. also used chlorophyll a (^1^O_2_ quantum yield is 0.77 in methanol) [32]. Cantrell et al. excited Bengal Rose ((^1^O_2_ quantum yield is 0.68 in ethanol) and (4-(1-pyrene)butyric acid) [33], like Ramel [34], to produce ^1^O_2_. Fiedor et al. used bacteriopheophytin-a (^1^O_2_ quantum yield is 0.2–0.3 in deuterated methanol) [35] and Scholz et al. 5,10,15,20-tetraphenyl-porphin (^1^O_2_ quantum yield is 0.60–0.70 in benzene) to evaluate β-carotene quenching [36]. Chanterell et al. also used another porphyrin, the protoporphyrin IX dimethyl ester ((^1^O_2_ quantum yield is 0.67 in dimethylsulfoxide (DMF)) [37]. In these published data, we observed a myriad of recorded *K_q_* values for investigated carotenoids. The differences between the values might be attributed to the different experimental conditions used for ^1^O_2_ production and detection. 

In our study, we decided to also choose a photosensitizer able to produce large amounts of ^1^O_2_, the porphyrin (P1COOH). Indeed, this molecule was synthesized by our group [25] and we found that the ^1^O_2_ quantum yield is 0.70 in ethanol and 0.87 in toluene. These quantum yields are similar to quantum yields of the photosensitizers used in the literature.

To detect ^1^O_2_ quenching, we recorded the decay of ^1^O_2_ in the presence of increasing amounts of carotenoids. In our study, all of the compounds were in hexane. We found a value similar to that of the literature (1.1 × 10^9^ M^−1^·s^−1^ in our case, 14 × 10^9^ M^−1^·s^−1^ in [38]). For lycopene, we found 1.1 × 10^9^ M^−1^·s^−1^ versus 19 × 10^9^ M^−1^·s^−1^ in [38]. The other carotenoids have not been studied in hexane so no comparison can be performed. The difference observed can be due to the different techniques used to evaluate ^1^O_2_ quenching. In [42], singlet oxygen was also detected via its luminescence at 1270 nm, but with a germanium photodiode linked to a Judson amplifier (Teledyne, Thousand Oaks, CA, USA) with a variable load resistor. In our case, we evaluated the lifetime of ^1^O_2_ with a single photon counting FluoroHub-B controller.

**A**ccording to our results, the rate constants of quenching of ^1^O_2_ by β-cryptoxanthin, β-carotene, and lycopene recorded in hexane showed that these pigments efficiently quench ^1^O_2_ production, with associated (Kq) values of 1.6 × 10^9^, 1.3 × 10^9^ and 1.1 × 10^9^ M^−1^·s^−1^. β-cryptoxanthin showed the higher quenching efficiency (1.6 × 10^9^ M^−1^·s^−1^). In organic solvents, carotenoids with 10 or 11 conjugated double bond quench singlet oxygens near the diffusion limit. β-cryptoxanthin, β-carotene, lycopene contained between 11 C=C and 13 C=C conjugated double bonds (Table 3), which explain the high Kq values obtained. The little differences in the rate constants between the three carotenoids in hexane can be explained by the similarity of the number of C=C conjugated double bonds for these carotenoids. The hydroxylated forms of carotenoids lutein, zeaxanthin, violaxanthin, 5,6-epoxy-α-carotene and the not identified xanthophyll (peak 4) revealed smaller quenching rate constants. This could be explained by a possible chemical reaction between ^1^O_2_ and the hydroxyl group of xanthophylls. Moreover, xanthophylls are well dissolved in polar solvents but not in non-polar solvents such as hexane. This might lead to aggregation and decrease of the quenching rate constants. 

## 4. Conclusions

Persimmon fruit cultivated in Tunisia is known for its richness in carotenoid pigments. In this study, it was possible to show that at least 11 carotenoids are present in the fruit, suggesting that persimmon fruit can be considered as a potential source of carotenoids. The photophysical properties (absorption, fluorescence, quenching of singlet oxygen) of carotenoids extracted from persimmon fruits and purified were investigated in hexane, using porphyrin as the photosensitizer for ^1^O_2_ production. Of the eight carotenoids investigated, β-cryptoxanthin, β-carotene and lycopene showed high quenching efficiencies compared to the xanthophylls (lutein, violaxanthin, zeaxanthin, 5,6-epoxy-α-carotene and the unidentified xanthophyll). The kinetic analysis of results obtained from the Stern–Volmer plot showed that β-cryptoxanthin quenches singlet oxygen as efficiently as β-carotene and lycopene, with *K_q_* values of 1.6 × 10^9^, 1.3 × 10^9^ and 1.1 × 10^9^ M^−1^·s^−1^. Xanthophylls in hexane gave lower values than 10^9^ M^−1^·s^−1^. The bimolecular rate constants of ^1^O_2_ quenching (*K_q_*) depend on the carotenoid structures such as the number of conjugated double bonds and the presence or absence of hydroxyl and epoxy groups. The efficiency of singlet oxygen quenching of β-cryptoxanthin can thus change the consideration that β-carotene and lycopene are the most singlet oxygen quenchers acting as catalysts for deactivation of the harmful ^1^O_2_. In the near future, photodynamic molecular beacons (PMB) using β-cryptoxanthin as ^1^O_2_ quencher will be synthesized and tested, hoping to show that natural molecules extracted from food matrices may be of interest for photodynamic therapy. 

## Figures and Tables

**Table 1:** Characteristics of carotenoids from persimmon obtained from preparative chromatography. λ_max_ and fine spectral data were observed on the UV-Vis spectrum of each collected carotenoid, in a powder form, and in petroleum ether.

Peak	Carotenoid	OH/Epoxy	t_R_ (min)	λ_max_ (nm) ^a^	% III/II ^a,b^	% AB/AII ^a,c^	[M + H]^+^ (*m*/*z*)
1	all-*trans*-violaxanthin	2/2	18	416/440/465 (416/440/465)	50 (98)	0	601
2	all-*trans*-lutein	2/0	23	420/443/469 (421/445/474)	25 (60)	0	569
3	all-*trans*-zeaxanthin	2/0	28	424/448/475 (424/449/476)	25 (25)	0	569
4	not identified (*ci*s-xanthophyll with 4 oxygens)	4	32	330/422/444/469	43	14	601
5	5,6-epoxy-α-carotene suspected	0/1	43	421/445/470 (418/441/469) *	0 (10) *	0	553
6	all-*trans*-*β*-cryptoxanthin	1/0	52	426/449/476 (425/449/476)	36 (25)	0	553
7	not identified (mixture of monoxygenated xanthophyll and carotene)	?	62	330/423/444/470	10	18	mixture 537/553
8	*cis*-isomere of *β-*carotene, position 13 (supposed)	0/0	67	332/421/443/470 (338/420/444/470) *	16 (12) *	20 (47) *	537
9	all-trans-*β-*carotene	0/0	74	426/449/476 (425/450/477)	23 (25)	0	537
10	*cis*-isomere of *β-*carotene, position 9 (supposed)	0/0	75	331/424/445/469 (330/420/444/472) *	11 (20) *	26 (18) *	537
11	all-*trans*-lycopene	0/0	86	444/470/502 (444/470/501)	66 (65)	0	537

^a^ values between brackets without * correspond to spectral characteristics also found in petroleum ether and compiled by Rodriguez-Amaya [28]. Values between brackets with * correspond to spectral characteristics along a methanol/ methyl-tert-butyl-ether (MTBE) gradient observed by De Rosso and Mercadante [29], who also used a C30 column; ^b^ ratio of the height of the longest-wavelength absorption peak, designated III, and that of the middle absorption peak, designated II, taking the minimum between the two peaks as baseline, multiplied by 100; ^c^ the relative intensity of the *cis*-peak is expressed as % AB/AII (Figure 3), which is the ratio of the height of the *cis*-peak, designated AB, and that of the middle main absorption peak, designated AII, multiplied by 100.

**Table 2:** K_q_ of carotenoids in different solvents, methods of ^1^O_2_ production and ^1^O_2_ quenching.

Carotenoids	k_Q_ 10^9^ M^−1^·s^−1^ (Solvent)	^1^O_2_ Production	Method of Detection of ^1^O_2_ Quenching	References
lycopene	17 (C_6_H_6_)	Phenazine	^1^O_2_ emission	[38]
	18 (C_6_H_5_CH_3_)	Phenazine	^1^O_2_ emission	[38]
	19(C_6_H_14_)	Phenazine	^1^O_2_ emission	[38]
	9.0 (CHCl_3_)	NDPO_2_	^1^O_2_ emission	[39]
	19 (CHCl_3_)	Phenazine	^1^O_2_ emission	[38]
	14 (CCl_4_)	Phenazine	^1^O_2_ emission	[38]
	13.8 (EtOH/CHCl_3_/D_2_O 50/50/1)	EP-1	DPBF	[40]
	31(EtOH/CHCl_3_/D_2_O 50/50/1)	NDPO_2_	^1^O_2_ emission	[41,42]
	8.8 (EtOH/CHCl_3_/D_2_O 50/50/1)	DMNO_2_	^1^O_2_ emission	[43]
	17.5(EtOH/CHCl_3_/H_2_O 50/50/1)	Phenazine	^1^O_2_ emission	[38]
	31 (EtOH/CHCl_3_/H_2_O 50/50/1)	NDPO_2_	^1^O_2_ emission	[41]
	0.13 (ascorbic acid in methanol)	1-NN	^1^O_2_ emission	[44]
	23–25 (reverse micelle (RM))	RB	DMA	[45]
	8.8 (CHCl_3_)	DMNO_2_	^1^O_2_ emission	[43]
	9 (EtOH/CHCl_3_/H_2_O 50/50/1)	NDPO_2_	^1^O_2_ emission	[46]
	6.93 (soybean oil)	Chlorophyll	Headspace oxygen depletion by gas chromatography	[47]
β-carotene	13.0 (C_6_H_6_)	Phenazine	^1^O_2_ emission	[38]
	13.8 (C_6_H_6_)	Anthracene/Naphthalene	Radiolysis/^1^O_2_ emission	[48]
	12.5–14 (C_6_H_6_)	Anthracene/Naphthalene	Radiolysis/^1^O_2_ emission	[49]
	13 (C_6_H_6_)	Anthracene	DPBF	[50]
	14 (toluene)	Phenazine	^1^O_2_ emission	[38]
	14 (C_6_H_14_)	Phenazine	^1^O_2_ emission	[38]
	5.0 (CHCl_3_)	NDPO_2_	^1^O_2_ emission	[39]
	14.0 (EtOH/CHCl_3_/H_2_O 50/50/1)	NDPO_2_	^1^O_2_ emission	[41]
	12 (EtOH/CHCl_3_/H_2_O: 50/50/1)	Phenazine	^1^O_2_ emission	[38]
	10.8 (EtOH/CHCl_3_/D_2_O 50/50/1)	EP-1	DPBF	[40]
	4.2 (EtOH/CHCl_3_/D_2_O 50/50/1)	NDPO_2_	^1^O_2_ emission	[41,42]
	8.4 (EtOH/CHCl_3_/D_2_O 50/50/1)	DMNO_2_	^1^O_2_ emission	[43]
	30 (MeOH/C_6_H_6_ 1/4)	MB	2-methyl-2-penten	[51]
	5 (MeOH/C_6_H_6_ 1/4)	Anthracene/Naphthalene	Radiolysis/^1^O_2_ emission	[52]
	13 (MeOH/C_6_H_6_ 1/4)	Anthracene/Naphthalene	Radiolysis/^1^O_2_ emission	[53]
	10.9 ± 0.5 (THF)	TPP	^1^O_2_ emission	[36]
	2.3 (DPPC)	RB and PBA	^1^O_2_ emission	[33]
	9.9 (CCl_4_)	Phenazine	^1^O_2_ emission	[38]
	5.9 (CCl_4_)	1H-P + RB	^1^O_2_ emission	[54]
	0.7 (CCl_4_)	Porphyrin	^1^O_2_ emission	[55]
	11 (CHCl_3_)	Phenazine	^1^O_2_ emission	[38]
	8.1 (CHCl_3_)	DMNO_2_	^1^O_2_ emission	[43]
	23 (C_6_H_6_/MeOH: 3/2)	MB/RB	^1^O_2_ emission	[56]
	1.5 (CD_3_OD)	1H-P/RB	^1^O_2_ emission	[54]
	0.35 (Ascorbic acid in MeOH)	1-NN	^1^O_2_ emission	[44]
	5 (H_2_O/ (CH_3_)_2_CO 12/88)	Riboflavin	GC with thermal conductivity	[57]
	12.67 (reverse micelle (RM))	RB	DMA	[45]
	5 (EtOH/CHCl_3_/D_2_O 50/50/1)	NDPO_2_	^1^O_2_ emission	[46]
Lutein	11.0 (C_6_H_6_)	TPP	^1^O_2_ emission	[58]
	16 (C_6_H_6_)	Phenazine	^1^O_2_ emission	[38]
	8.0 (EtOH/CH_2_Cl_2_/H_2_O; 50:50:1)	NDPO_2_	^1^O_2_ emission	[41]
	0.11 (DPPC)	RB and PBA	^1^O_2_ emission	[33]
	9.24 (EtOH/CHCl_3_/D_2_O 50/50/1)	EP-1	DPBF	[40]
	2.4 (EtOH/CHCl_3_/D_2_O 50/50/1)	NDPO_2_	^1^O_2_ emission	[42]
	21 (MeOH/C_6_H_6_ 1/4)	Anthracene/Naphthalene	Radiolysis/^1^O_2_ emission	[53]
	1.3 (ascorbic acid in methanol)	1-NN	^1^O_2_ emission	[44]
	10-33 reverse micelle (RM)	RB	DMA	[45]
	5.72 (soybean oil)	Chlorophyll	Headspace oxygen depletion by gas chromatography	[47]
β-cryptoxanthin	7.31 (EtOH/CHCl_3_/D_2_O 50/50/1)	EP-1	DPBF	[40]
	1.8–6 (EtOH/CHCl_3_/D_2_O 50/50/1)	NDPO_2_	^1^O_2_emission	[41,42]
Zeaxanthin	12.6 (C_6_H_6_)	Phenazine	^1^O_2_emission	[38]
	12 (C_6_H_6_)	Phenazine	^1^O_2_emission	[38]
	2.8 (C_6_H_6_)	Anthracene/Naphthalene	Radiolysis/^1^O_2_ emission	[48]
	10 (EtOH/CHCl_3_/D_2_O 50/50/1)	NDPO_2_	^1^O_2_emission	[41]
	0.23 (DPPC)	RB and PBA	^1^O_2_emission	[33]
	10.5 (EtOH/CHCl_3_/D_2_O 50/50/1)	EP-1	DPBF	[40]
	3.0 (EtOH/CHCl_3_/D_2_O 50/50/1)	NDPO_2_	^1^O_2_emission	[41]
	10 (EtOH/CHCl_3_/H_2_O 50/50/1)	NDPO_2_	^1^O_2_emission	[41]
	0.77 (ascorbic acid in methanol)	1-NN	Radiolysis/^1^O_2_ emission	[44]
	6.79 (soybean oil)	Chlorophyll	Headspace oxygen depletion by gas chromatography	[47]
Violaxanthin	16 (C_6_H_6_)	Phenazine	^1^O_2_ emission	[38]
	9 reverse micelle (RM)	RB	DMA	[45]

Car: carotenoid; DHIR: -3,3-dihydroxyisorenieratene; DMA: 9,10-dimethylanthrancene; DMNO_2_: 1,4-dimethyl-1,4-naphthalene endoperoxide; DPBF: 1,3-Diphenylisobenzofuran; EP-1: 3-(1,4-epidioxy-4-methyl-1,4-dihydro-1-naphtyl propionic acid); EtOH: ethanol; Hex: hexane; 1-HP: 1H-Phenalen-1-one; MB: methylene blue; NDPO_2_: 3,3′-(1,4-naphthalylene dipropionate); 1-NN: (1-nitronaphtalene); PBA: 4-(1-pyrene)butyric acid; RB: Rose Bengal; RM: system of sodium (bis-2-ethylhexanyl)sulfosuccinate in (hexane/H_2_O); TPP: tetraphenylporphyrin.

**Table 3:** Bimolecular rate constants *K_q_* of the quenching of ^1^O_2_ by carotenoids from persimmon fruits in hexane.

Carotenoids	Nb of C=C and OH Group	*K_q_* (M^−1^·s^−1^)
β-carotene in hexane	11 C=C	1.1 × 10^9^
β-cryptoxanthin	11 C=C and 1 OH	1.6 × 10^9^
lycopene	13 C=C	1.1 × 10^9^
Lutein	11 C=C and 2 OH	8.0 × 10^8^
Zeaxanthin	11 C=C and 2 OH	6.0 × 10^7^
(Peak 4)	-	7.2 × 10^7^
5,6-epoxy-α-carotene	10 C=C and 1 OH, 2 epoxy	3.8 × 10^7^
Violaxanthin	9 C=C, 2 OH and 2 epoxy group	5.8 × 10^7^

## Abbreviations

APCI	atmospheric pressure chemical ionization
ASE	accelerated solvent extraction
BHT	butylhydroxytoluene
Car	carotenoid
DHIR	3,3-dihydroxyisorenieratene
DMA	9,10-dimethylanthrancene
DMNO2	1,4-dimethyl-1,4-naphthalene endoperoxide
DPBF	1,3-Diphenylisobenzofuran
EP-1	3-(1,4-epidioxy-4-methyl-1,4-dihydro-1-naphtyl propionic acid)
EtOH	ethanol
Hex	hexane
1-HP	1H-Phenalen-1-one
LC-MS	liquid chromatography- mass spectrometry
LDL	low-density lipoprotein
MB	methylene blue
MTBE	methyl-tert-butyl-ether
NDPO_2_	3,3′-(1,4-naphthalylene dipropionate)
1-NN	(1-nitronaphtalene)
PBA	4-(1-pyrene)butyric acid
P1COOH	5-(4-carboxyphenyl)-10,15,20-triphenylporphyrin
PDT	photodynamic therapy
PMB	photomolecular beacons
RB	Rose Bengal
RM	system of sodium (bis-2-ethylhexanyl)sulfosuccinate in (hexane/H_2_O)
TEA	triethylamine
TPP	tetraphenylporphyrin
